# The NBS Two-Pressure Humidity Generator, Mark 2

**DOI:** 10.6028/jres.081A.010

**Published:** 1977-02-01

**Authors:** S. Hasegawa, J. W. Little

**Affiliations:** Institute for Basic Standards, National Bureau of Standards, Washington, D.C. 20234

**Keywords:** Calibration, dew point, humidity, hygrometer, mixing ratio, relative humidity, two-pressure generator, volume ratio, water vapor

## Abstract

A new humidity calibration facility which uses the two-pressure principle for generating gas of known humidity has been developed at NBS for calibrating and testing hygrometers. The relative humidity range of the two-pressure humidity generator is 3 to 98 percent for ambient temperatures −60 ° to 80 °C and test chamber pressures 5 to 200 kPa (absolute). This is equivalent to a nominal dew/frost point range of −80 ° to 80 °C. Intercomparison tests were made with the NBS standard gravimetric hygrometer over a portion of the generator’s operating range. The estimated maximum uncertainty (three standard deviations) is 0.2 percent *RH* for temperatures 0 ° to 80 °C which in units of dew point corresponds to an estimated maximum uncertainty of 0.04 °C for dew points −35 ° to 80 °C.

## 1. Introduction

The National Bureau of Standards develops, maintains, and disseminates the standards for the humidity measurement system in the United States of America. A gravimetric hygrometer serves as the primary standard [1]^1^. Two precision humidity generators [2, 3] are used as the principal facilities for calibrating transfer and secondary standards, and for testing and evaluating hygrometers and sensors. One of these generators [2] has been in use since 1951. It operates on what is now known as the two-pressure principle. A stream of gas at an elevated pressure is saturated with respect to the liquid or solid phase of water and then expanded to a lower pressure. Measurements of the pressure and temperature of the saturated gas stream, and in the test chamber after expansion, yield the data necessary to compute the water vapor content of the gas stream. By selecting and maintaining appropriate temperatures and pressures, it is possible to generate any desired level of humidity in the gas stream. Over the past 25 years this generator has performed with reliability, convenience, and versatility. When the decision was made to replace this old generator with a modern facility, the inherent advantages of the two-pressure principle were the basis for the new design. It seems appropriate, therefore, to call the new generator “The NBS Two-Pressure Humidity Calibration Facility, Mark 2.”

Mark 2 produces a continuous stream of gas which can flow at any specified rate up to 0.005 m^3^/s – through a test chamber in which the relative humidity can be varied from 3 to 98 percent, the temperature from **−**60 ° to 80 °C and the absolute pressure from 5 to 200 kPa. It is designed to operate either manually or with digital computer control. This paper describes the operation in the manual mode with the gas stream in the test chamber at approximately 100 kPa.

## 2. General Considerations

The calibration of a hygrometer with the two-pressure humidity generator can be made in various units which relate to the quantity of water vapor in a moist gas. Among the most common units are mixing ratio, dew-point temperature, relative humidity, and volume ratio. These expressions are defined in terms of real gas behavior and account for the fact that the saturation pressure of pure water in the presence of an inert gas differs from that of pure water alone. These units have been defined in general terms by Harrison [4]. Wexler [5] has defined these units explicitly for the two-pressure generator in terms of the measurements of the saturation temperature and pressure (i.e., in the final saturator) and the temperature and pressure in the test chamber or other test space.

The saturation mixing ratio, *r_w_*, of the moist gas emerging from the generator is

$$r_{w}=\frac{M_{w}f(P_{s},T_{s})e_{w}(T_{s})}{M_{g}[P_{s}-f(P_{s},T_{s})e_{w}(T_{s})]}$$ (1)

where

*M_w_*: = molecular weight of water vapor,
*M_g_*: = molecular weight of the carrier gas,
*e_w_*(*T_s_*): = saturation vapor pressure over a plane surface of the pure phase of liquid or solid water at the saturator temperature, *T_s_*,
*P_s_*: = saturator pressure and
*f*: = enhancement factor and is defined below.

The enhancement factor, *f*, at the saturator pressure, *P_s_*, and temperature, *T_s_*, is expressed by

$$f(P_{s},T_{s})=\frac{x_{w}P_{s}}{e_{w}(T_{s})}=\frac{(1-x_{g})P_{s}}{e_{w}(T_{s})}$$ (2)

where

*x_g_, x_w_*: = the mole fractions of gas and water vapor in the saturated mixture, respectively.

The definition of the relative humidity *RH* in the test chamber of the generator is $RH={\left(x_{v}/x_{w}\right)}_{P_{c},T_{c}}\times 100$

where

*x_v_*: = the mole fraction of water vapor in a given sample of moist air characterized by pressure, *P_c_*, and temperature, *T_c_*, and
*x_w_*: = the mole fraction of water vapor in the saturated mixture at the same values of pressure, *P_c_*, and temperature, *T_c_.*

Substituting appropriate expressions for the mole fractions yields

$$RH=\frac{f(P_{s},T_{s})}{f(P_{c},T_{c})}\times \frac{e_{w}(T_{s})}{e_{w}(T_{c})}\times \frac{P_{c}}{P_{s}}\times 100$$ (3)

where

*f*(*P_c_, T_c_*): = enhancement factor at test chamber pressure, *P_c_*, and temperature, *T_c_*,
*e_w_*(*T_c_*): = saturation vapor pressure over a plane surface of the pure phase of liquid or solid water at the test chamber temperature. *T_c_*, and
*P_c_*: = test chamber pressure.

The “thermodynamic dew-point (or frost-point) temperature” *T_d_* of a moist gas at absolute total pressure, *P*, is defined as that temperature at which the moist gas is saturated with respect to a plane surface of pure liquid (or solid) water. The dew point, *T_d_*, of the moist gas of the two-pressure generator is obtained by the iterative solution of

$$f(P_{c},T_{d})e_{w}(T_{d})=f(P_{s},T_{s})e_{w}(T_{s})\frac{P_{c}}{P_{s}}$$ (4)

where *e_w_* and *f* are values obtained from suitable tables and *P_c_* is the absolute pressure in any space or volume that is filled with the moist gas, e.g., the mirror chamber of a dewpoint hygrometer.

The volume ratio, *V*, of the moist gas of the two-pressure generator is

$$V=\frac{f(P_{s},T_{s})e_{w}(T_{s})}{P_{s}-f(P_{s},T_{s})e_{w}(T_{s})}.$$ (5)

Hence, with the establishment of constant temperatures and pressures in the saturator and test chamber, the various units of humidity can be calculated for the moist gas produced by the two-pressure humidity generator. The formulations^2^ of Wexler and Greenspan [6] and Goff [7] are used for obtaining the saturation vapor pressure of water and ice, respectively. When air is used as the carrier gas, the values for the enhancement factor, *f*, for air, given by Hyland [8], over the temperature range −50 ° to 90 °C and pressures from 0.25 × 10^5^ to 10^7^ Pa are used in the above equations. Greenspan [9] has obtained a simplified equation for *f* which can be easily programmed for a computer or can be calculated with the aid of a programmable pocket calculator.

## 3. Description

**Figure 1:**
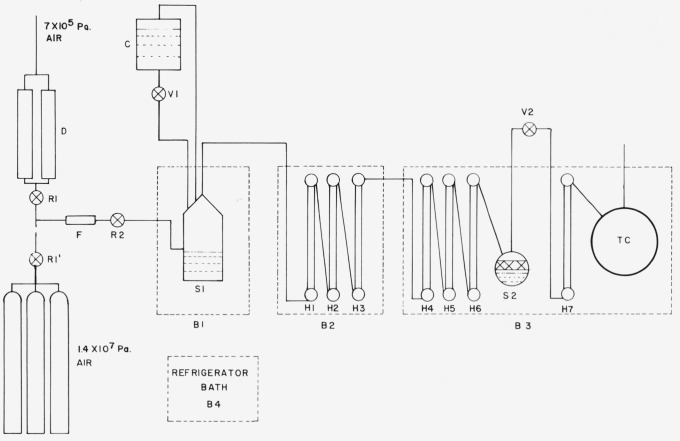
Schematic diagram of two-pressure humidity generator.
DAir drier and filtersR1, R2Pressure regulatorsFFlowmeterS_1_PresaturatorB_1_Presaturator bathV_1_Water supply control valve for presaturatorCWater reservoir for presaturatorB_4_Refrigeration bath for cooling baths B_1_, B_2_ & B_3_B_2_Conditioning bathH_1_–H_6_Radiator type heat exchangerB_3_Final bathS_2_Final saturatorV_2_Digital expansion valveH_7_Heat exchanger coilTCTest chamber

The two-pressure method for generating or producing air of known humidity involves saturating the air or other gas at high pressure with water vapor and then expanding the air to a lower pressure. The generator is designed for a maximum saturator operating pressure of 3.3 MPa which in turn controls the minimum relative humidity. Figure 1 is a simplified flow diagram which illustrates the principle of operation and the basic components.

Compressed air from the house pressure line is first cleaned by using commercially available filters and air driers, D. Alternatively, in the case where a gas other than air is used or air of higher pressure than available from the house air line is required, cylinders of high pressure gas are connected to a manifold and the gas is introduced into the apparatus downstream of the desiccant towers. Two pressure regulators, R_1_ and R_2_, are used to control the pressure in the humidifying system and a flowmeter, F, is used to monitor the flow rate.

Saturation of the air at high pressure is accomplished in two saturators, S_1_ and S_2_. Presaturator, S_1_, is immersed in a bath, B_1_, which is maintained at a temperature of 10 ° to 15 °C warmer than the desired saturation temperature. The presaturator utilizes a centrifugal flow pattern. Air enters the presaturator tangentially to the inner wall and directed slightly downward into the water. A liquid level controller automatically maintains a fixed water level in the presaturator by controlling the solenoid valve, V_1_. Supply water is maintained in a container, C, which is at the same pressure as the presaturator. The air enters the presaturator through a coil of 1.27 cm (0.5 in) o.d. tubing and exits at the top of the saturator through a 5.08 cm (2 in) o.d. tubing. Types 316 or 304 stainless steel tubing and fittings are used throughout the system.

The air then passes through the three heat exchangers. H_1_, H_2_ and H_3_, located in the bath, B_2_, which is operated at a temperature of 0.5 ° to 1.0 °C warmer than the final saturation temperature. The air which enters the heat exchangers in bath, B_2_, is warmer than and also supersaturated with respect to the temperature of bath, B_2_. The purpose of bath, B_2_, is to make it easier to achieve temperature control in the final bath, B_3_, by tempering the air so that it is close to, but still slightly above, the final saturation temperature.

**Figure 2:**
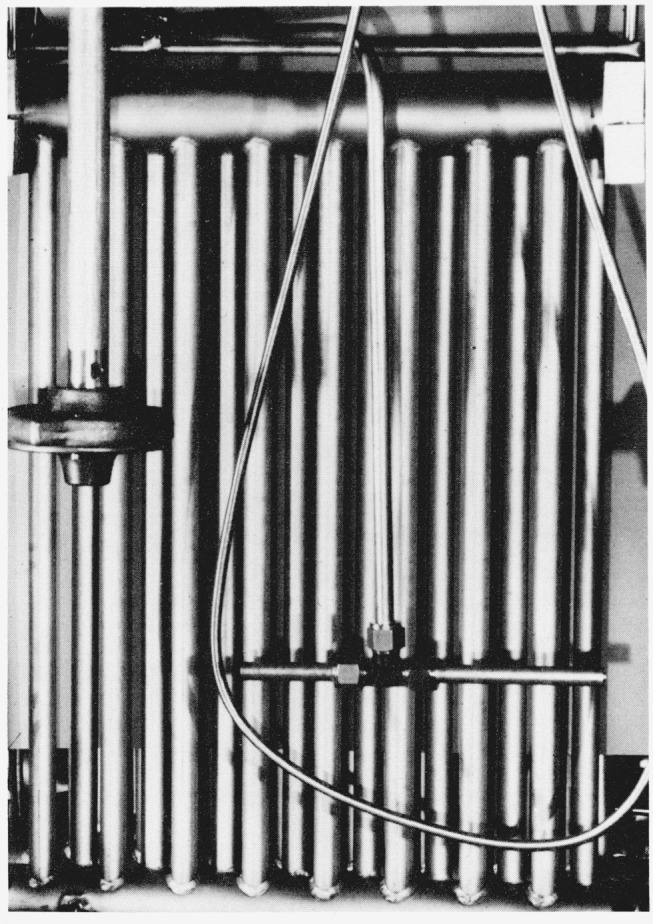
Radiator type heat exchanger

The six radiator type heat exchangers, H_1_ through H_6_ located in baths, B_2_ and B_3_, were designed for minimum pressure drop. The air enters the bottom of each heat exchanger which is a horizontal tube 5.08 cm (2 in) o.d. and 0.5 m (18 in) in length. The top of the heat exchanger, which is similar in design and dimensions to the bottom, is connected to the bottom with sixteen parallel tubes 1.9 cm (0.75 in) o.d. and 0.5 m (20.5 in) in length as shown in figure 2.

After the air emerges from the conditioning bath, B_2_, it is brought to the final saturation value by flowing through the three heat exchangers, H_4_–H_6_ and the final saturator, S_2_, which are located in the bath, B_3_. Under the worst operating conditions, i.e., at a flow rate of 0.005 m^3^/s and at a pressure of one atmosphere, the maximum pressure drop between the presaturator, S_1_, and the outlet of the final saturator, S_2_, is 300 Pa. Although the pressure drop in the three heat exchangers in the final bath, B_3_, is small, there is still a finite pressure drop and if this pressure drop occurs after the air reaches the final saturation temperature, it will cause the air to be unsaturated. To make up this deficit, a final saturator, S_2_, is installed downstream of the heat exchanger, H_6_. In addition to resaturating the air, the final saturator provides additional surface area to assure that any water droplets which may be entrained in the air stream precipitate out.

**Figure 3:**
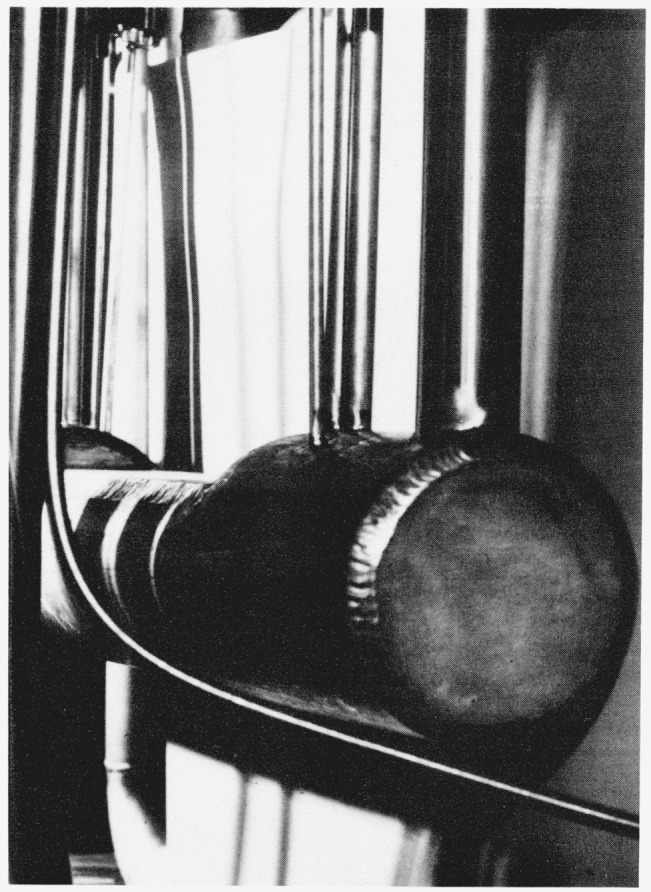
Final Saturator

The final saturator, S_2_, is a tube having an o.d. of 11.43 cm (4.5 in) with wall thickness of 0.95 cm (0.375 in) and a length of 66.0 cm (26.0 in). The tube is placed in a horizontal position and is half filled with water. A segment from the top of the tube was removed and replaced with a flat plate so that the height of the air space above the water is 2.5 cm. In addition, the air stream is divided into three channels above the water surface and in each channel there is a maze of air deflectors to force the air onto the water surface. In figure 3, which is a photograph of the final saturator, the large tube located near the end of the saturator is the air outlet tube. The other, smaller tubes provide access for pressure and temperature measurements and also a tap through which the saturated air can be brought from the generator at the saturator pressure, by-passing the expansion valve, V_2_, and the test chamber, *T_c_.*

**Figure 4:**
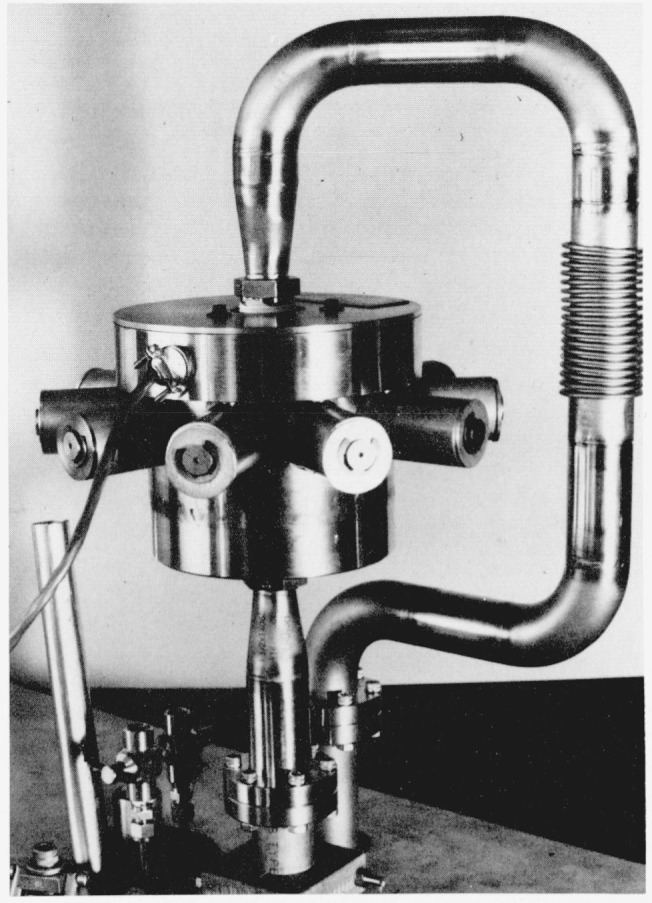
Expansion valve

Upon emerging from the final saturator, S_2_, the air is expanded to a lower pressure through an expansion valve, V_2_, figure 4, which is placed external to the bath, B_3_. This valve is thermally insulated and provided with a heater to maintain the valve and plumbing above the dew-point temperature of the adiabatically cooled test gas. The expansion valve is a digital control valve with a 9-bit resolution. It controls ten flow elements (nozzles) whose effective total cross-sectional areas can be chosen over a range of 511 to 1. The effective areas of the flow control elements are arranged in a binary sequence. The valve has a maximum valve coefficient, *C_v_*^3^, of 5 and a resolution of approximately 0.2 percent or a *C_v_* of 0.01.

**Figure 5:**
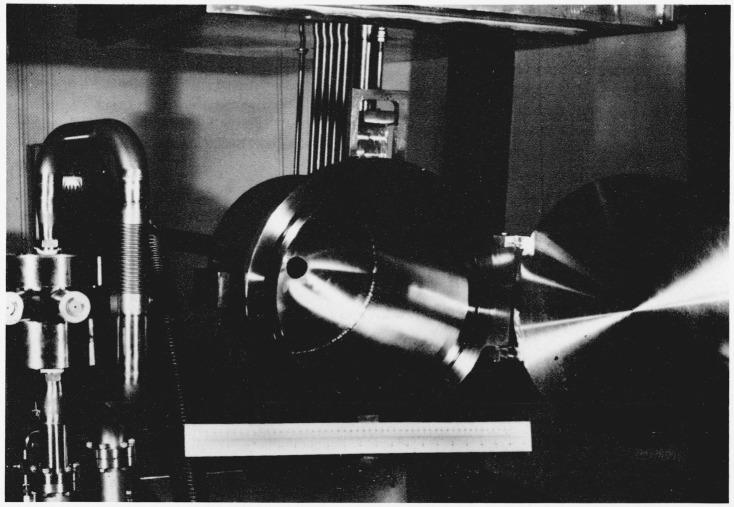
Test chamber

After expansion, the air passes through the heat exchanger, H_7_, which is a coil of tubing, 3.8 cm (1.5 in) o.d. and approximately 11 m in length, to bring the air back to the bath temperature before it enters the test chamber, TC, figure 5. The air from the test chamber discharges into the laboratory or into a vacuum source. In the former case, the test chamber will remain at or near atmospheric pressure; in the latter, the test chamber can be controlled at sub-atmospheric pressures through the use of a suitable back-pressure regulator. A length of flexible stainless steel hose between the test chamber and the final heat exchanger permits the test chamber to be taken out of the bath. The test chamber is a cylinder 32.4 cm (12.75 in) o.d., 0.95 cm (0.374 in) wall thickness and 40.6 cm (16 in) in length. Tubular outlets extend from the chamber to allow mechanical controls and electrical leads to be brought in and out of the working space and for measuring the temperature and pressure inside the chamber.

As stated previously the temperature of the final bath, B_3_, can be maintained over the range −60 ° to 80 °C. The temperatures of the baths, B_1_, B_2_, and B_3_, are each maintained by balancing a small amount of constant cooling with controlled heating. Cooling is induced by pumping the liquid bath fluid from each bath through its associated heat exchange coils located in the refrigeration bath, B_4_, which in turn is cooled with liquid carbon dioxide. The thermocouple indicator and temperature controller for bath, B_4_, is an on-off type which controls the opening and closing of a solenoid valve on a liquid carbon dioxide line. The temperature controllers for baths, B_1_ and B_2_, are the power proportional type with resistance thermometer sensor and for bath, B_3_, time proportional with reset using a platinum resistance thermometer sensor.

## 4. Instrumentation

As indicated in eqs 1 through 5, the calculations of the various units of humidity require the measurement of the temperatures and pressures of the final saturator and the test chamber.

To facilitate the automation of data acquisition, the resistances of the calibrated four-lead standard platinum resistance thermometer and the calorimetric type platinum resistance thermometer are measured with a 385 Hz excited bridge based on a design by Cutkosky [10]. The bridge utilizes an inductive ratio divider and requires only one adjustment for balancing. A built-in phase-sensitive null-detector easily resolves 1 *μ*Ω in 25 Ω. Small deviations from balance are recorded continuously with an analog recorder and/or the BCD output of a digital voltmeter is used for recording the data on a teletypewriter at any preselected time interval. The platinum resistance thermometers were calibrated at NBS on the International Practical Temperature Scale of 1968 and subsequently checked from time to time at the triple point of water. It is estimated that the uncertainty in the temperature measurement is an order of magnitude more accurate than the required 10 millidegrees.

The pressures are measured with calibrated fused quartz Bourdon tube pressure gages equipped with BCD outputs. The ranges of the pressure gages used in the generator are 0 to 0.21 MPa (30 psia) for the test chamber and 0 to 0.69 MPa (100 psia) or 0 to 3.3 MPa (500 psig) for the saturator. These gages are periodically calibrated with a dead weight piston gage. The accuracy of the pressure measurements is estimated at 70 Pa.

## 5. Performance

Two independent approaches were used to evaluate the performance of this generator. First, an intercomparison was made between the generator and the NBS gravimetric hygrometer. Second, an analysis was made of all known possible sources of error and from this analysis, an estimate was derived for the accuracy that could be expected from the generator.

### 5.1. Intercomparison Tests

A 3 × 3 Graeco-latin square experiment [11] was used to test four variable parameters of the two-pressure humidity generator. The experiment was designed to determine whether any of the preselected levels of the parameters could affect the accuracy of the generator. The four parameters which were tested in the experiment were the presaturator temperature, the final saturator temperature, the pressure of the saturator, and the test air flow. Three levels for each of the four parameters were used in the test.

The parameters were arranged in the following form:

**Table:** 

*T*_−20_, Δ*t*_0_, *P*_2_, *F*_10_	*T*_0_, Δ*t*_5_, *P*_5_, *F*_10_	*T*_25_, Δ*t*_15_, *P*_1_, *F*_10_
*T*_−20_, Δ*t*_5_, *P*_1_, *F*_5_	*T*_0_, Δ*t*_15_, *P*_2_, *F*_5_	*T*_25_, Δ*t*_0_, *P*_5_, *F*_5_
*T*_−20_, Δ*t*_15_, *P*_5_, *F*_1_	*T*_0_, Δ*t*_0_, *P*_1_, *F*_1_	*T*_25_, Δ*t*_5_, *P*_2_, *F*_1_

where

*T*: = the final saturator temperature and *T*_−20_ = −20 °C, T_0_ = 0 °C, *T*_25_ = 25 °C;
Δ*t*: = the difference in the temperature between the presaturator and the final saturator, the subscript indicating the amount in degrees Celsius that the former exceed the latter;
*P*: = the final saturator pressure and *P*_1_ = 10^5^ Pa, *P*_2_ = 2 × 10^5^ Pa, *P*_5_ = 5 × 10^5^ Pa; and
*F*: = the rate of test air flow and *F*_1_ = .03 m^3^/min, *F*_5_ = .15 m^3^/min, *F*_10_ = .3 m^3^/min (at 25 °C and 10^5^ Pa).

Each box of the Graeco-latin square represents a run and the four parameters were maintained at the designated levels. The mixing ratio of the moist air produced by the two- pressure generator was calculated by using eq (1) for each of the runs and the results were compared with the value of the mixing ratio as measured by the NBS standard hygrometer [1]. The NBS standard hygrometer has a maximum uncertainty of 0.12 percent.

## a. Results

A percentage difference, *d*, was obtained by using the eq *d* **=** (*r_w_ – r_g_*)*/r_g_* × 100 where *r_w_* is the computed mixing ratio for the generator and *r_g_* is the mixing ratio measured by the NBS standard hygrometer. The results of the tests for the 3×3 Graeco-latin square experiment are given in table 1. Two values in a box represent a repeat run.

In the first column of table 1, *T*_−20_ is the value of the parameter, *T*, common to all three boxes in that column while the other three parameters are each represented at the three different levels indicated. Similarly, *T*_0_ and *T*_25_ are the values of the parameter, *T* common to all boxes in columns two and three, respectively. If the four tested parameters are assumed to be independent of each other and have no interactions, then the average value in each column is indicative of the correlation of the percentage difference, *d*, with the value of *T* for that column. Similar analyses of the rows yield the correlation of *d* with *F*, analyses of one set of diagonals give the correlation of *d* with *P*, while analyses of the second set of diagonals give the correlation of *d* with Δ*t*. These results are given in table 2.

In order to assess the significance of *d* in table 2, it is necessary to remember that the maximum uncertainty in *r_g_* is 0.12 percent. Therefore, if *d* exceeds 0.12 percent, the increase is ascribed to *r_w_*, and, more particularly, to the corresponding parameter level.

### 5.2. Error Analysis

An estimate of the maximum uncertainty in the calculated mixing ratio, *r_w_*, of the generator for each of the runs was obtained by using the estimated systematic uncertainty plus three times the random uncertainty of the measured pressure and temperature in the final saturator. The maximum uncertainty of the enhancement factor, which also includes the uncertainty in the saturation vapor pressure values, was obtained from table 9 of Hyland’s [8] paper. The standard deviations were computed for the measured temperature and pressure of the saturator in each of the runs and the standard deviation of the mean was used as a measure of the random uncertainty. The systematic uncertainty in the pressure measurement was attributed to the uncertainty in the calibration of the pressure gage, and for the temperature measurement, the systematic uncertainty was primarily due to the maximum temperature gradient detected in the final bath which contains the saturator and the test chamber.

The estimated uncertainty in the calculated mixing ratio was obtained from the expression

$$\frac{\Delta r_{w}}{r_{w}}=\frac{P_{s}}{P_{s}-fe_{w}}{\left[{\left(\frac{\Delta e_{w}}{e_{w}}\right)}^{2}+{\left(\frac{\Delta P_{s}}{P_{s}}\right)}^{2}+{\left(\frac{\Delta f}{f}\right)}^{2}\right]}^{1/2}.$$ (6)

Table 3 lists the estimated systematic uncertainty and the 3*σ* errors for each of the runs and also the estimated maximum uncertainty of the generator.

By inserting the values of the estimated maximum uncertainty given in table 3 in the appropriate squares of the 3×3 Graeco-latin square and by computing the mean for each row, column and diagonal of the square, the results obtained are a measure of the estimated maximum uncertainty of the generator for each level of the tested parameters. These results are given in table 4.

Comparison of the results (to the nearest tenth of a percent) of tables 2 and 4 show that the calculated maximum uncertainty of the generator is equal to or greater than the percentage difference *d* for the various levels of the tested parameters and therefore it may be concluded that the performance of the generator is not affected by the four tested parameters over the range which these parameters were tested.

## 6. Accuracy

The results of the intercomparison tests of the generator with the standard hygrometer which are shown in the previous section indicate that the estimated maximum uncertainty of the generator (based on the systematic and 3*σ* uncertainties of the temperature and pressure measurements and the maximum error for the enhancement factor) are equal to or greater than the measured difference, *d.* Therefore, similar calculations were made beyond the range covered by the intercomparison tests to obtain the estimates of the maximum uncertainty for the generator over the temperature range of −55 ° to 80 °C and for pressures from ambient to 3.3 × 10^6^ Pa. Table 5 lists these results which are given in units of mixing ratio, volume ratio, dew-point temperature and relative humidity.

## 7. Conclusion

The NBS Two-pressure Humidity Calibration Facility, Mark 2 was designed for manual or computer operation and to encompass a humidity range of −80 ° to +80 °C dew points. The generator was also designed to operate over the ambient pressure range of 5000 to 2 × 10^5^ Pa and temperature range of 80 ° to −55 °C.

Intercomparison tests were made with the NBS Standard Hygrometer (gravimetric) over a limited range of the generator. The results of the tests showed that the difference between the humidity calculated for the generator based on measurements of pressure and temperature and the humidity measured by the standard hygrometer were equal to, or less than, the estimated uncertainties based on the estimates of the maximum contributions to the systematic uncertainty plus three times the standard deviation for the parameters of the pressure, temperature and enhancement factor. The estimates of the maximum uncertainty of the generator are listed in table 5. The magnitude of the uncertainties for the new generator is at least 50 percent less than current estimated uncertainties used for humidity at the National Bureau of Standards [3].

Work is still in progress on the development of an interface between the generator and a minicomputer and on testing the generator for subatmospheric pressure operation. It is also planned to extend the intercomparison tests with the standard hygrometer at both higher and lower humidities.

## Figures and Tables

**Table 1:** The percentage difference between r_w_ and r_g_

−0.30% (*T*_−20_, Δ *t*_0_, *P*_2_, *F*_10_)	+ 0.10% (*T*_0_, Δ *t*_5_, *P*_5_, *F*_10_)	+ 0.10% (*T*_25_, Δ *t*_15_, *P*_1_, *F*_10_)
+ 0.17%, −0.32% (*T*_−20_, Δ *t*_5_, *P*_1_, *F*_5_)	+0.09% (*T*_0_, Δ *t*_15_, *P*_2_, *F*_5_)	+0.08% (*T*_25_, Δ *t*_0_, *P*_5_, *F*_5_)
−0.12% (*T*_−20_, Δ *t*_15_, *P*_5_, *F*_1_)	−0.14% (*T*_0_, Δ *t*_0_, *P*_1_, *F*_1_)	+0.09%, +0.22% (*T*_25_, Δ *t*_5_, *P*_2_, *F*_1_)

**Table 2:** The correlation of parameter levels with the percentage difference between r_w_ and r_g_

*d*		*d*		*d*		*d*	
*T*_−20_	0.23%	Δ*t*_15_	0.10%	*P*_5_	0.10%	*F*_10_	0.17%
*T*_0_	0.11%	Δ*t*_5_	0.18%	*P*_2_	0.18%	*F*_5_	0.17%
*T*_25_	0.12%	Δ*t*_0_	0.17%	*P*_1_	0.18%	*F*_1_	0.14%

**Table 3:** Experimental errors

Saturator		Estimated systematic errors		3*σ*-Random errors		$\frac{\Delta e_{w}}{e_{w}}$	$\frac{\Delta P_{s}}{P_{s}}$	$\frac{\Delta f}{f}$^a^	Quad^b^ $\frac{\Delta r_{w}}{r_{w}}\times 100$
Temp.	Press.	Temp.	Press.	Temp.	Press.				

(°C)	(Pa)	(°C)	(Pa)	(°C)	(Pa)				(%)
−20	2 × 10^5^	0.030	69.0	0.00042	41.4	0.00293	0.00055	0.00085	0.31
−20	1 × 10^5^	0.030	69.0	0.00048	47.6	0.00293	0.00117	0.00045	0.32
−20	1 × 10^5^	0.030	69.0	0.00045	18.6	0.00293	0.00088	0.00045	0.31
−20	5 × 10^5^	0.030	69.0	0.00033	24.8	0.00293	0.00019	0.00210	0.36
0	5 × 10^5^	0.010	69.0	0.00012	33.1	0.00073	0.00020	0.00160	0.18
0	2 × 10^5^	0.010	69.0	0.00009	9.0	0.00073	0.00039	0.00065	0.11
0	1 × 10^5^	0.010	69.0	0.00009	16.5	0.00073	0.00086	0.00035	0.12
25	1 × 10^5^	0.010	69.0	0.00033	22.8	0.00060	0.00092	0.00015	0.11
25	5 × 10^5^	0.010	69.0	0.00012	24.8	0.00060	0.00019	0.00088	0.11
25	2 × 10^5^	0.010	69.0	0.00018	8.3	0.00060	0.00039	0.00038	0.08
25	2 × 10^5^	0.010	69.0	0.00042	18.6	0.00060	0.00044	0.00038	0.08

a Enhancement factor uncertainties obtained from Hyland’s [8] paper.

b Combined by quadrature, i.e., the square root of the sum of the squares.

**Table 4:** The estimated maximum uncertainty of the generator’s mixing ratio for the designated levels of the tested parameter

*T*_−20_	0.33%	Δ*t*_15_	0.19%	*P*_5_	0.22%	*F*_10_	0.20%
*T*_0_	0.14%	Δ*t*_5_	0.19%	*P*_2_	0.15%	*F*_5_	0.21%
*T*_25_	0.10%	Δ*t*_0_	0.18%	*P*_1_	0.22%	*F*_1_	0.16%

**Table 5:** NBS two-pressure humidity generator, mark 2, range and accuracy

Humidity parameter	Range	Accuracy^a^

Mixing ratio, *r_w_* (g water vapor/kg dry air)	0.0005 ≤ *r_w_*< 0.0015	3.0% of value
	0.0015 ≤ *r_w_*< 0.005	1.5% of value
	0.005 ≤ *r_w_*< 0.1	1.0% of value
	0.1 ≤ *r_w_*< 0.3	0.5% of value
	0.3 ≤ *r_w_*< 515	0.3% of value
Volume ratio, *V* (ppm)	1 ≤ *V*< 3	3.0% of value
	3 ≤ *V*< 10	1.5% of value
	10 ≤ *V*< 170	1.0% of value
	170 ≤ *V*< 500	0.5% of value
	500 ≤ *V*< 820,000	0.3% of value
Dew-point temperature, *T_d_ °*C	−80 ≤ *T_a_*< −70	0.2
	−70 ≤ *T_d_* < −35	0.1
	−35 ≤ *T_d_* < +80	0.04
Relative humidity, *RH* (%) at test chamber temperature *T_c_* (°C) of:		
−55 ≤ *T_c_*< −40	3–98	1.5
−40 ≤ *T_c_*< −20	3–98	0.8
−20 ≤ *T_c_*< 0	3–98	0.4
0 ≤ *T_c_*< +80	3–98	0.2

a The estimated bounds to systematic error plus three times the standard deviation.

## Footnotes

1 Figures in brackets indicate the literature references at the end of this paper.

2 In the near future the formulations by Wexler, “Vapor Pressure Formulation for Water in the Range 0 ° to 100 °C. A Revision” and “Vapor Pressure Formulation for Ice,” both to be published in the J. Res. Nat. Bur. Stand. (U.S.), will be used. Since Hyland’s [8] values for enhancement factor *f* for air are dependent on the vapor pressure formulation, the enhancement factor will be modified to conform to the new values of the vapor pressure. However, the use of the new values of vapor pressure and enhancement factor will make only very small changes in the final results since the magnitude of the product *f* × *e_s_* must remain the same.

3 The value coefficient, *C_v_*, is the number of gallons of water at 60 °F that will pass through the valve per minute with a pressure drop of 1 lb/in^2^.
